# Comparison of Dimensionality Reduction Methods in Mass Spectra of Astrocytoma and Glioblastoma Tissues

**DOI:** 10.5702/massspectrometry.A0094

**Published:** 2021-03-13

**Authors:** Evgeny Zhvansky, Anatoly Sorokin, Vsevolod Shurkhay, Denis Zavorotnyuk, Denis Bormotov, Stanislav Pekov, Alexander Potapov, Evgeny Nikolaev, Igor Popov

**Affiliations:** 1 Moscow Institute of Physics and Technology, Dolgoprudny, Moscow Region, Russian Federation; 2 Institute of Cell Biophysics RAS, Pushchino, Russian Federation; 3 Institute of Systems, Molecular and Integrative Biology, University of Liverpool, Liverpool, UK; 4 Federal State Autonomous Institution «N.N. Burdenko National Scientific and Practical Center for Neurosurgery» of the Ministry of Healthcare of the Russian Federation, Moscow, Russian Federation; 5 N.N. Semenov Federal Research Center of Chemical Physics Russian Academy of Sciences, Moscow, Russian Federation; 6 Skolkovo Institute of Science and Technology, Moscow, Russian Federation

**Keywords:** mass spectra, astrocytoma, glioblastoma, brain tumors, feature selection, dimensionality reduction

## Abstract

Recently developed methods of ambient ionization allow the collection of mass spectrometric datasets for biological and medical applications at an unprecedented pace. One of the areas that could employ such analysis is neurosurgery. The fast *in situ* identification of dissected tissues could assist the neurosurgery procedure. In this paper tumor tissues of astrocytoma and glioblastoma are compared. The vast majority of the data representation methods are hard to use, as the number of features is high and the amount of samples is limited. Furthermore, the ratio of features and samples number restricts the use of many machine learning methods. The number of features could be reduced through feature selection algorithms or dimensionality reduction methods. Different algorithms of dimensionality reduction are considered along with the traditional noise thresholding for the mass spectra. From our analysis, the Isomap algorithm appears to be the most effective dimensionality reduction algorithm for negative mode, whereas the positive mode could be processed with a simple noise reduction by a threshold. Also, negative and positive mode correspond to different sample properties: negative mode is responsible for the inner variability and the details of the sample, whereas positive mode describes measurement in general.

## INTRODUCTION

It is well known that the key parameter, defining life expectancy for patients with a brain tumor is the excess of tumor resection since the tumor cells could provoke a relapse.^1)^ There are a number of methods for tumor boundary detection, such as MRI,^2)^ PET,^3)^ fluorescence,^4,5)^ ultrasound,^6)^ *etc.*, however, all of them have their own limitations. Recently we observe a growing interest in applications of mass spectrometry for tumor tissue identification, typing, and tumor boundary detection.^7–9)^ Analysis of tumor samples by mass spectrometry is based upon the observation that tumor cells substantially differ from normal ones in metabolic processes and as consequences have different chemical content.^10)^ Identification of histological type and localization of brain tumor tissue during neurosurgery allows the correctness of tumor dissection and paves the way for the personalized strategy of further patient treatment using chemotherapy taking into account molecular characteristics of the tumor. The comparative analysis of tumor types has fundamental value, though the clarification of tumor borders has the highest priority from the neurosurgeons’ point of view. The most important problem of the neurosurgery assistive tool is tumor cells detection in the transition zone between tumor and unmodified tissue, which is necessary for clinical application of the mass spectrometric methods for intraoperative tumor border monitoring. The better tumor border detection—the lower probability of the relapse and the highest median survival.^11)^

Analysis of the high-dimensional mass spectrometric (MS) data usually employs dimensionality reduction (DR) algorithms as the preliminary step for statistical analysis and visualization. Among DR methods most widely used are linear methods such as PLS-DA and PCA.^12–17)^ Recently more advanced non-linear methods, for example, t-SNE and UMAP to name a few, were developed.^18)^ However, even the trivial thresholding operation could be used as a DR approach. We have demonstrated that thresholding allows the construction of a feature set of manageable size and applying this feature set for the successful identification of different tissue types.^19)^ Another area where DR algorithms are of high demand is hyperspectral imaging such as MS imaging. It is common for MS imaging data analysis to visualize data in pseudocolors when each RGB channel of the pixel is defined by the value of three selected feature’s values. The first three PCA components and three selected ions are the most commonly used features in such visualization.^20,21)^

In this paper, we compare the performance of two linear, five nonlinear DR algorithms and thresholding in their ability to emphasize the differences between mass spectra of two histological classes of objects. Inspired by the MS imaging approach we also used a visualization technique called the spectra similarity matrix (SSM) to compare different approaches performance. SSM allows rapid visual evaluation of similarity of a large set of spectra as was previously shown^22)^ (Fig. S1).

We have used astrocytoma and glioblastoma MS data obtained with inline cartridge extraction^23)^ as a dataset. Each sample of the tumor is characterized by a series of consecutively measured mass spectra (scans) during the extraction. The key difficulty in the analysis of this dataset is that both inter and intra class variability is extremely high. The intraclass variability could be attributed to differences in patients, tumor location, and intratumor morphological variations in the tissues dissected during single neurosurgery. These intratumor variations could be explained by the tumor genesis processes,^10)^ so the tumor could be represented as a combination of benign and malignant or more and less benign part of the tumor.^24)^ The high variability may cause problems for rapid, precise, and objective analysis of the tumor-specific features. Adequate selection of DR algorithm for MS data processing is necessary for the confident application of statistical and machine learning techniques for the development of intraoperative tumor border monitoring techniques.

## EXPERIMENTAL

### Measurements

Spectra were measured in low resolution under clinical conditions with Thermo LTQ XL. Inline cartridge extraction^23)^ followed by electrospray ionization was used for mass spectrometric profiling of samples, which proceeds microextraction of substances from the sample in the solvent flow, while the sample is held on the glass microfiber filter. Spectra from Thermo LTQ XL were measured in both positive and negative ion modes with resolutions 2000 at *m*/*z* 744. All spectra were measured in the *m*/*z* 100–2000 ranges. The exact description of the experimental protocol was published previously.^25)^

### Samples

Tissue samples of 86 glioblastoma tissues from 30 patients and 76 astrocytic tissues from 26 patients were provided by the N.N. Burdenko NSPCN and analyzed under a protocol approved by N.N. Burdenko NSPCN Institutional Review Board (order 40 from 12.04.2016, revised with order 131 from 17.07.2018). A signed informed consent form, filled out in accordance with the requirements of the local ethical committee, specifically noting that all removed tissues can be used for further research, was obtained from all patients before surgery. The study was conducted in accordance with the Helsinki Declaration as revised in 2013. All procedures were carried out according to the relevant guidelines and regulations.

According to histology analysis dataset contains 30 glioblastomas (WHO Grade IV; 9 with IDH-1 R132H mutation) with different IDH status, 17 anaplastic astrocytomas (WHO Grade III; 8 tumors with IDH-1 R132H mutation), 8 diffuse astrocytomas (WHO Grade II; 6 tumors with IDH-1 R132H mutation) and 1 gemistocytic astrocytoma (WHO Grade II with IDH-1 R132H mutation).

Usually, 3 fragments from each dissected tissue sample from the same single patient were measured with each instrument for taking into account and evaluating inner biological variability.

One part of each tissue sample was annotated with routine hematoxylin and eosin staining and further immunohistochemical analysis of tissue fragments. Three other fragments of each sample were measured with Thermo LTQ XL right after neurosurgery.

### Processing

Spectra were processed with the algorithm as follows. Mass spectra were binned with bin width 0.25 *m*/*z* as described previously.^26)^ Spectra of each measurement were filtered by a moving median filter with width and step equal to 51. SSM with cosine measure similarity was calculated as described previously^22)^ (Fig. S1). The baseline subtraction was carried out through an algorithm described previously.^27)^

### Dimensionality reduction

DR was made with Scikit-learn^28)^ machine learning library using default parameters values provided for each method if not specified explicitly. Two linear (PCA and partial least squares discriminant analysis (PLS-DA)^29)^) and four nonlinear (non-negative matrix factorization (NNMF),^30)^ isometric mapping (Isomap),^31)^ UMAP^32)^ and Diffusion map^33)^) methods were used for DR to three-, five- and six-dimensional data representation. For the thresholding the levels of threshold were selected to keep 5, 10, 25, 75, and 200 highest intensities in each scan.

Linear methods looking for a linear combination of features (intensities) of mass spectra that are able to capture the most important properties of the set of spectra and reduce the dimensionality of the data by rejecting noisy and low-intensity components of such representation. So the intensities are always linearly taken into account for output calculation and that makes results of such calculations easy to interpret and up to some extent reversible, which means it is possible to find which points in the original dimension correspond to a particular point in low-dimensional representation. Linear methods work rather well when groups of spectra are linearly separable, which means there is a hyperplane such that each group located on one side of it. Nonlinear methods allow distinguishing the groups of spectra that are not linearly separable, for example, form a set of concentric circles. Nonlinear methods are difficult to interpret and extract information about the contribution of particular feature intensity into the position of the spectra in low-dimensional representation. PCA produces linear combinations of features as components of the highest variability in the data. NNMF converts the matrix (M) into a product of nonnegative factors W*H so that the factors W and H minimize the root mean square residual between M and W*H. So, W is the matrix of coordinates (columns) of the components H (rows) in the new representation. PLS-DA linearly separates data into components that have the most distinguishing power to given labels. Isomap provides components of a better representation of the internal structure (geometry) of the data based on the mutual Euclidean distance between raw features of the data. Diffusion map provides a low-dimensional representation of the data while keeping its local geometry based on the graph of the data and diffusion distances calculated from Markov processes for the data. UMAP—uniform manifold approximation and projection algorithm that allows the different scale of the data to be highlighted, so both local and large-scale data structures could be observed through the components of UMAP. The number of nearest neighbors was chosen to five for UMAP and Isomap algorithms, as the contrast for five components is optimal in data representation (Figs. S19–S22). Higher values lead to lower contrast of SSMs or rough structure data representation.

All calculations and visualizations were made using code written by the authors using MATLAB and Python. The code is available on request. The time required for algorithms to be calculated on the dataset (808 spectra and 7600 features in each) is up to one minute.

## RESULTS

Dimensionality reduction is an important step in the analysis of mass spectrometry data, which is required for visualization for further application of mathematical, statistical, and machine learning techniques. The ideal DR algorithm should preserve both global and local structures in the original data and at the same time denoise data. Dimensionality needs to be reduced to two or three dimensions for visualization purposes because only such data could be analyzed by human beings. Many statistical and mathematical methods prefer that the number of features was less than the number of samples. In medical mass spectrometry datasets that requirements never fulfill as the number of peaks could easily be in hundreds or thousands, while the number of samples rarely exceeds 100. In our dataset, spectra are located in 7600-dimensional space.

The most widely used DR techniques in mass spectrometry are classical linear methods such as PCA and PLS-DA.^12–17)^ Recently a number of new DR algorithms were developed, such as NNMF,^30)^ isometric mapping (Isomap),^31)^ UMAP.^32,34)^ We exclude the famous t-SNE^35)^ algorithm and recently published PHATE^6)^ from consideration in this paper, as they require the whole dataset to generate the mapping and does not allow calculating projection of new points afterward, which is necessary for methods to be used with machine learning techniques down the pipeline.

Thresholding is usually applied to remove noise from the signal. During thresholding intensity set to zero for all bins, which have original intensity below the specified value. This procedure could be also considered as a naive method of DR, in which only a subset of high-intensity bins forms dimensions in projection space. So we calculated transformed spectra, in which only 5, 10, 25, 75, and 200 highest intensities are preserved in each spectrum. This procedure creates spaces dimensions which are shown in Table 1.

**Table 1:** Table 1. Dependence between threshold value (the number of intensities in each spectrum) and non-zero bins in the average spectrum of all spectra.

N peaks	Dimensionality	
	Negative	Positive
5	120	72
10	204	130
25	403	332
75	897	789
200	1687	1592
7600	7553	7543

From this data, it could be seen that spectra in the positive mode are more conservative because the dimensionality of the data grows slower, which means that on average the same bins have high intensity in spectra more often in positive than in negative mode. So, keeping only 5 major peaks in each spectrum we could reduce data dimensionality by two orders of magnitude.

### Visual analysis

**Figure 1:**
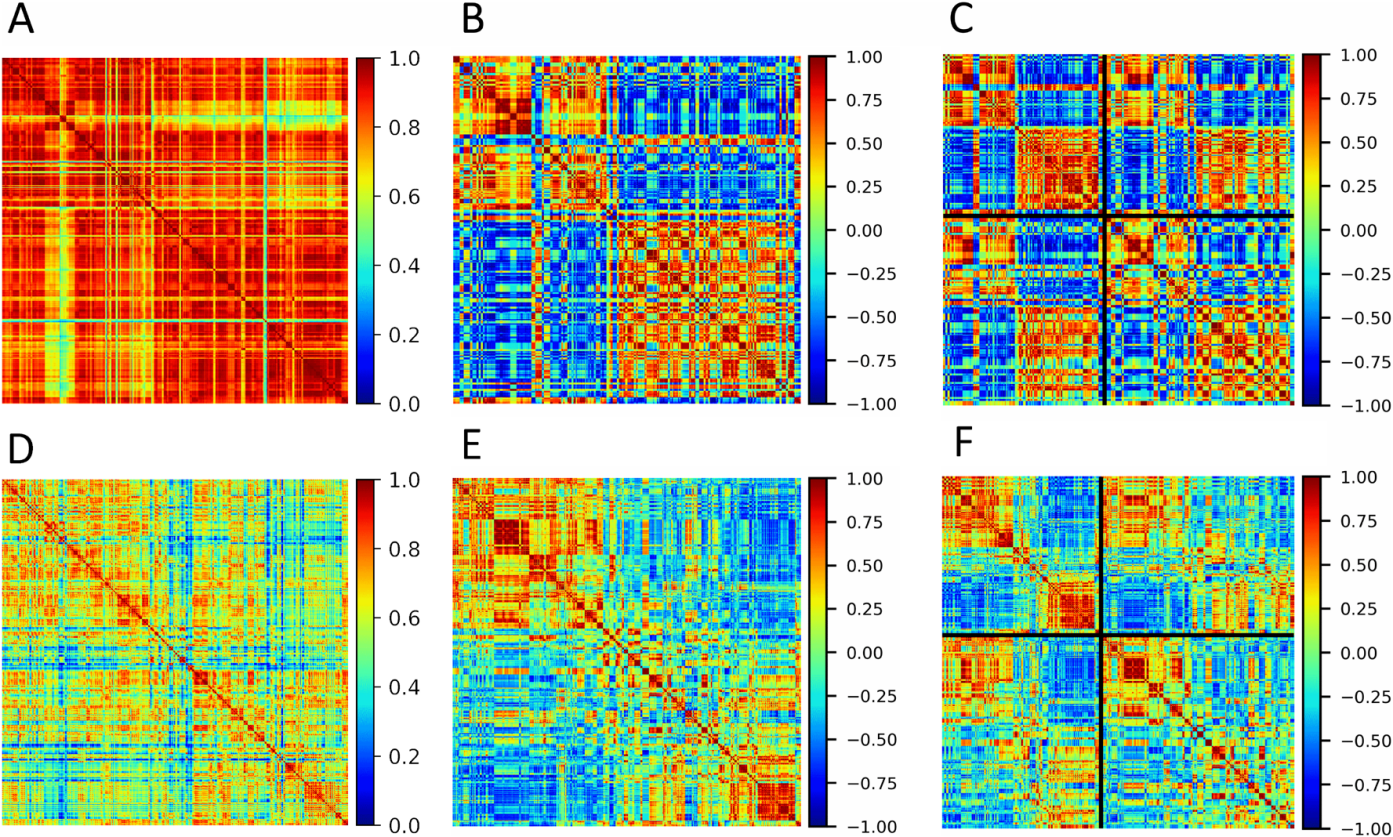
Fig. 1. SSM images for the Isomap DR application. Top row (A, B, C) - positive mode, bottom row (D, E, F) - negative mode. Left column (A, D) - raw spectra, middle column (B, E) - SSM for 7 features after Isomap DR ordered by date of acquisition, right column (C, F) - SSM for 7 features after Isomap DR ordered by diagnosis. Take into account that colormap axis has different limits in the left column.

Recently we have developed a fast and efficient visualization method for evaluation stability and reproducibility of the mass spectra called SSM.^22)^ In the nutshell SSM is the matrix, every cell of each contains a value of similarity measure between two spectra, corresponding to indices of column and row of matrices in Fig. 1. Any measure could be used for SSM construction, here we are using cosine measure for the computational performance reasons. A well-known correlation matrix is a particular case of SSM in which correlation is used as (dis)similarity measure and it is widely applied in mass spectrometry^36)^ and other disciplines.^37)^

Here we adopt SSM analysis for the evaluation of DR approaches performance. The visual analysis here is based on the ability of the human eye to detect structure in properly colored images. From this point of view, feature selection techniques and DR methods in particular should increase contrast in properly organized SSM images. When spectra of the same class are located next to each other, properly selected features should keep low intraclass while increasing inter-class similarity. Visually it turned out as two squares of high-value cells located on the main diagonal of the matrix, separated by low values off-diagonal areas. A great example of such contrast is Isomap in Figs. 1B and 1E, where we can see two distinct classes represented by red squares. In Figs. 1C and 1F spectra are organized differently and we can see that two classes are split into four smaller subsets along the main diagonal. In addition to these small red squares, we can see off-diagonal red rectangles indicating the presence of groups of highly similar spectra located far from each other along the matrix border.

### Positive mode data

It was shown already that different modes of spectra emphasize different characteristics of samples.^25,38)^ Data from positive mode is more stable as we have seen in Table 1, and characterizes the data acquisition process.

First, we have analyzed the results of thresholding (Figs. S2 and S3). For each threshold value, we have made two SSM plots. In the first plot, SSM scans are arranged according to the date of acquisition (Fig. S2). In this SSM we could check the presence of the so-called ‘batch effect,’ which corresponds to changes in measurement conditions due to device maintenance. In the second plot, SSM scans are first aligned according to diagnosis and within the diagnosis scans aligned according to the date of acquisition (Fig. S3). The boundary between diagnoses is shown as vertical and horizontal black lines. This plot should help us to detect differences between classes of data, which is a diagnosis in our case.

**Figure 2:**
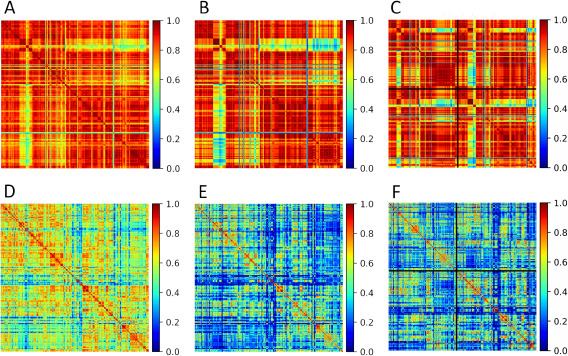
Fig. 2. SSM images for the dataset with thresholding. Top row (A, B, C) - positive mode, bottom row (D, E, F) - negative mode. Left column (A, D) - raw spectra, middle column (B, E) - SSM for thresholding (5 and 25 features) ordered by date of acquisition, right column (C, F) - SSM for thresholding (5 and 25 features) ordered by diagnosis.

It could be seen that in positive mode SSM hardly changes when we decrease the dimensionality of data from 7600 to 72 (Figs. 2A and 2B). The batch effect is almost unnoticeable in Figs. 2B and S2, however, when we align data according to diagnosis in Figs. 2C and S3, it becomes visible, as the astrocytoma group is visually split into two more homogeneous subgroups (Fig. S18).

**Figure 3:**
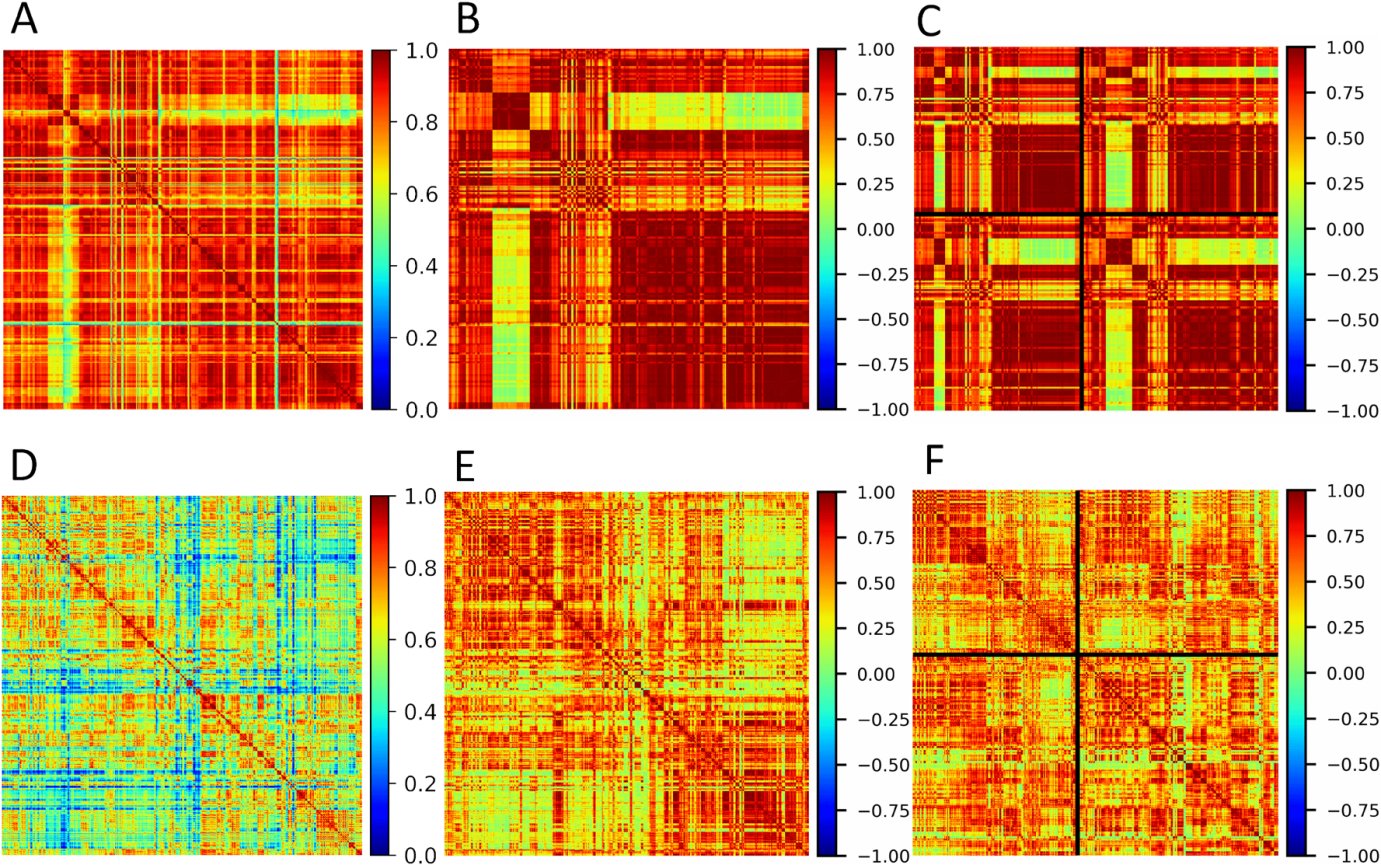
Fig. 3. SSM images for the dataset with NNMF DR. Top row (A, B, C) - positive mode, bottom row (D, E, F) - negative mode. Left column (A, D) - raw spectra, middle column (B, E) - SSM for NNMF (3 and 7 features) ordered by date of acquisition, right column (C, F) - SSM for NNMF (3 and 7 features) ordered by diagnosis.

**Figure 4:**
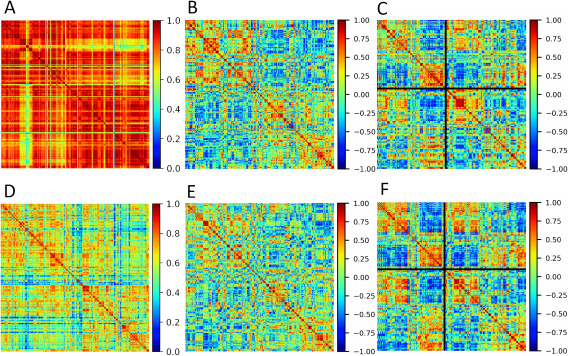
Fig. 4. SSM images for the dataset with PLS-DA DR. Top row (A, B, C) - positive mode, bottom row (D, E, F) - negative mode. Left column (A, D) - raw spectra, middle column (B, E) - SSM for PLS-DA 7 features ordered by date of acquisition, right column (C, F) - SSM for PLS-DA 7 features ordered by diagnosis.

When we apply DR algorithms to positive data, the batch effect becomes more visible (Figs. S6 and S7). Please note that the range of colormap on DR figures Figs. S6–S17 is different from thresholding figures Figs. S2–S5. NNMF (Fig. 3) and Isomap (Fig. 1) gave the best visualization of the batch effect in Fig. S6. On PCA, Diffusion map, and PLS-DA (Fig. 4) it is noticeable. Surprisingly both versions of UMAP gave no visual manifestation of batch effect. Figure S7 also shows that UMAP is not able to contrast differences between diagnoses also. Such a bad performance of a well-recognized algorithm could be explained by using default values for all parameters except distance measure. We know that the results of UMAP are quite sensitive to parameters such as minimal distance ε and number of neighbors μ. A proper selection of these parameters is required for each dataset.

We have varied the dimensionality of the target space in the output of DR methods, so the SSMs were calculated for 3 (Figs. S8 and S9), 5 (Figs. S6 and S7), and 7 (Figs. S10 and S11) compressed components. It could be seen that NNMF and Isomap are more contrast in Figs. S8 and S9, when the dimension is 3, while neither batch nor diagnosis effect is visible on PCA and PLS-DA in this dimension. The diffusion map happens to be least sensitive to the variation of the dimensionality of the target space in positive mode.

### Negative mode data

Data from the negative mode is much less stable than in the positive mode as could be seen from Figs. S4 and S5. We have discussed previously that negative data controls fine variations between samples, patients, and diagnoses.^25,38)^ Indeed, the batch effect is much less contrast in Fig. S5 compared to Fig. S3. It is also clear from Figs. S4 and S5 that contrary to the positive mode thresholding has a profound effect on data in negative mode. In 120-dimensional space, constructed by leaving only 5 top intensities in the spectra, the overall similarity on SSM is below 0.2. That means that the top 5 intensities rarely have the same position and the same value in different spectra. The structure of the SSM is gradually improved once we increase the dimensionality of data and become almost indistinguishable when we preserve 22% of original bins by keeping 200 top intensities in each spectrum.

When we applied DR methods to the negative mode spectra, we observed that at low target dimensions of 3 (Figs. S14 and S15) and 5 (Figs. S12 and S13) NNMF became useless as they were not able to contrast neither batch nor diagnosis effect. The only method that gave reliable contrast on SSM in the case of the target dimension of 3 was Isomap. Both linear algorithms (PCA and PLS-DA) and Diffusion map start performing better from the target dimension 5 (Figs. S12 and S13). NNMF somehow recovers its performance on the target dimension 7 (Figs. S16 and S17) but contrary to the positive mode data even in this case contrast provided by NNMF is worse than one from PCA.

It is interesting enough, that neither positive nor negative data did not show a noticeable diagnosis difference, which proves that analysis of the difference between glial tumors requires careful rational feature selection which could not be achieved ever by sophisticated DR algorithms. On the other hand, the presence of such global variation in the spectra as the batch effect could be easily detected by the application of DR techniques in both modes and by thresholding in the positive mode. The best performance was demonstrated by the Isomap algorithm, while NNMF is useful mainly in positive mode, which is characterized by a much more stable spectrum structure.

PCA does not produce any satisfying results for both positive and negative mode: there are no meaningful structures revealed in 3, 5, or 7 components (Figs. S6–S17). PCA dimensionality reduction leads to blurring the contrast parts of the SSM view. Thus, PCA does not reproduce the internal structure of the data.

## DISCUSSION

The large dimensionality of the mass spectrometry data causes problems for further analysis. Most statistical methods that use Gaussian distribution become less efficient in feature space with dimensions above 10–12, as at those dimensions the shape of a multidimensional Gaussian is almost indistinguishable from a multidimensional sphere. Many machine learning techniques suffer from the “curse of dimensionality” when an algorithm quickly becomes intractable with an increase in the number of feature space dimensions. In medical mass spectrometry, a small number of samples also requires a reduction of the dimensionality of the dataset. All of the above makes DR techniques an important step in the mass spectrometry processing pipeline. In this paper, we compared the performance of seven DR algorithms with each other and with a naive approach of DR by thresholding the spectra by the number of major peaks.

The analysis of results of the simple thresholding approach demonstrates the high level of homogeneity of SSM in the positive mode and great variability of SSM in the negative mode while varying the threshold, which corresponds to the stability of the spectra structure in positive and their variability in negative mode. The structure of the data is easy to observe in the positive mode with any threshold and proper spectrum ordering. At the same time, there is no obvious structure in the data in the negative mode until thresholding preserves 20% of original features. This could be explained by the differences in spectra characteristics in different polarities. In the positive mode, major intensities (about ten highest) are rather stable and their relative intensity does not vary too much. Contrary to this in the negative mode spectra, the major peaks have significant variability of the relative intensities. This leads to the situation when almost all values in SSM are zero for a high threshold in negative mode, despite the fact that the dimensionality in negative mode is almost 50% higher when five major intensities are preserved. This peak intensity variability could also map to the high variability of cosine measure in SSM when a higher number of peaks is selected. Hence, one could suppose that the positive mode better describes the measurement in general, whereas spectra in the negative mode better reflect details of the particular sample. This conclusion could be important as it suggests the need for design approaches for the combined analysis of both polarities of each sample in the dataset. To conclude, the thresholding procedure seems to be reasonable in positive mode for tracking global changes in the structure of the spectra due to variation in measurement procedures or sample processing. In the negative mode, thresholding could help only for the denoising of the spectra.

A comparison of the DR algorithms demonstrates that the Isomap algorithm outcompetes all other methods in both modes and all three selected target dimension values. This algorithm is not widely used in mass spectrometry and is usually mentioned together with the much more popular t-SNE in the analysis of mass spectrometry imaging. Isomap highlights the major groups of the data in a most contrastive way and the groups of the data have low dispersion in the visual analysis of the corresponding SSMs, which means it should reduce an error rate for the following data analysis techniques.

Another algorithm that performs very well was NNMF, which is also known for its applications in mass spectrometry imaging. The benefit of NNMF is the non-negative nature of its loading, which makes interpretation of the NNMF component much more straightforward in comparison to PCA and PLS. The non-negative values of the components also make it visually different from other methods. However, it demonstrates clear detection of the batch-effect in the positive mode, while its performance in negative mode is worse than of Isomap.

Together with PCA, PLS-DA is one of the most widely used algorithms of dimensionality reduction. PLS-DA is the only considered supervised algorithm in this paper, which takes into account target labels. PLS-DA demonstrates similar results in negative and positive modes. So, the SSM views for both polarities are similar and demonstrate the visible batch-effect (Figs. S6–S17). However, the comparison of the PLS-DA results with other algorithms results reveals a rather good representation of the data structure for the positive mode and significantly worse representation for the negative mode. Surprisingly, the PLS-DA demonstrated worse results than Isomap for the positive mode, though in the negative mode their results were comparable. This could be explained if we assume that the positive mode is responsible for the experimental specifics, whereas the negative mode is responsible for the specifics of the sample and patients.

UMAP is a very promising method, which has numerous adjustable parameters that allow its application to absolutely different data types taking into account the specifics of the dataset in a concrete case. The UMAP in our case did not succeed. We think this is due to the use of the default parameter set. The parameters should be adjusted in the future for using UMAP for dimensionality reduction and using its results with classifiers.

In general, five components of reduced space of features for positive mode and seven components for negative mode are enough for the detection of structure in the data and specifics of the spectra by most analyzed algorithms, which is suitable for further analysis with machine learning and statistical data exploration algorithms.

Previously the thresholding by peaks amount reveals opportunity for feature set selection in classification tasks,^19)^ however, in that work a specially designed feature selection procedure was applied to the data after thresholding. Current analysis proves that the additional processing is necessary for extraction information required for interpreting the data. It also proves that the global alterations in spectrum structure, caused by changes in measurement procedure, such as different sources of solutions or device maintenance, could be easily detected by a simple combination of dimensionality reduction and correlation analysis in reduced space. Contrary to that diagnosis- patient- and sample-specific features of the spectra require more elaborate feature selection supervised procedures.

The presented method is not directly applicable to the imaging data, however, it is inspired by MS imaging, which uses components of the reduced feature space to visualize MS imaging structures. We think that imaging mass spectrometry could employ this method eliminating information about the spatial structures. The spectra would be aligned in order to keep spatial closeness, and mutual similarity will be calculated, which could lead to understanding the number of classes and important features in reduced feature space. But detailed analysis is beyond the scope of the current paper.

## CONCLUSION

The positive and the negative modes characterize the measurement differently. Both polarities should be measured for a large description of the sample by its mass spectra, measured without sample preparation (ambient-like ionization methods). The positive mode is less variative and is better for batch-effects demonstrating. The negative mode is more variative and is better for annotating the specific sample.

The most effective algorithm for dimensionality reduction is Isomap. Further optimization of hyperparameters should be carried out for a better choice of dimensionality reduction algorithm. Nevertheless, the Isomap algorithm reveals the best performance in the information completeness of stayed features. The Isomap demonstrates the best contrast for batch-effect even for the negative mode, where spectra have a rather high-intensity variability, which minimizes the batch-effect presence. UMAP might be more effective than the Isomap algorithm, but it is the open question of the hyperparameter optimization.

## Notes

Mass Spectrom (Tokyo) 2021; 10(1): A0094
