# Supplementary material for: Comparison of Dimensionality Reduction Methods in Mass Spectra of Astrocytoma and Glioblastoma Tissues
Source: Mass Spectrom (Tokyo). 2021 Mar 13;10(1):A0094. doi: 10.5702/massspectrometry.A0094 (PMC7953827; doi:10.5702/massspectrometry.A0094)
Supplement: Supplementary Data [file massspectrometry-10-1-A0094_s001.pdf]

The spectra similarity matrix (SSM) is a matrix filled with the values of the mutual similarity of all scans in analyzed measurements (Fig. S1).

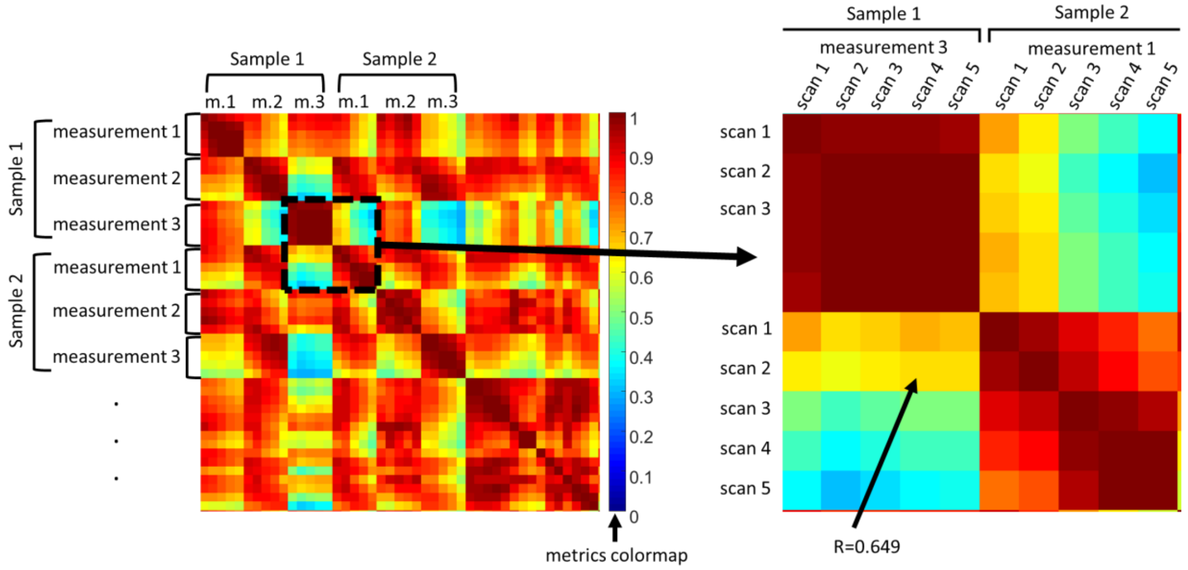

Figure S1. Correlation matrix between mass spectra of different samples and their fragments on the left. Scans are grouped by measurements and ordered by time from left to right and from top to bottom within the measurement. Selected area is shown on the right at the individual pixel scale. Correlation coefficient R=0.649 is pointed for the second scan of the first measurement of the second sample and the fourth scan of the third measurement of the first sample.

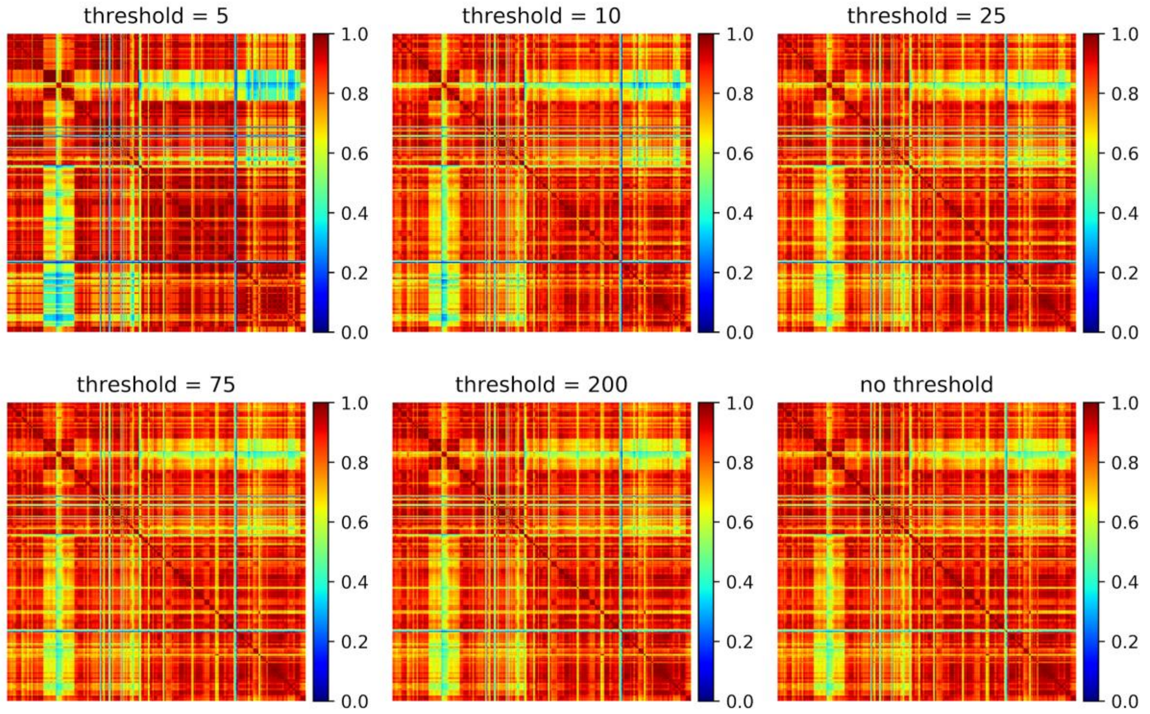

Figure S2. SSM for spectra sorted by measurement time with different filtering thresholds - the amount of the most intensive peaks in each spectrum. Spectra measured under following conditions: positive mode, low-resolution, 100-2000 m/z.

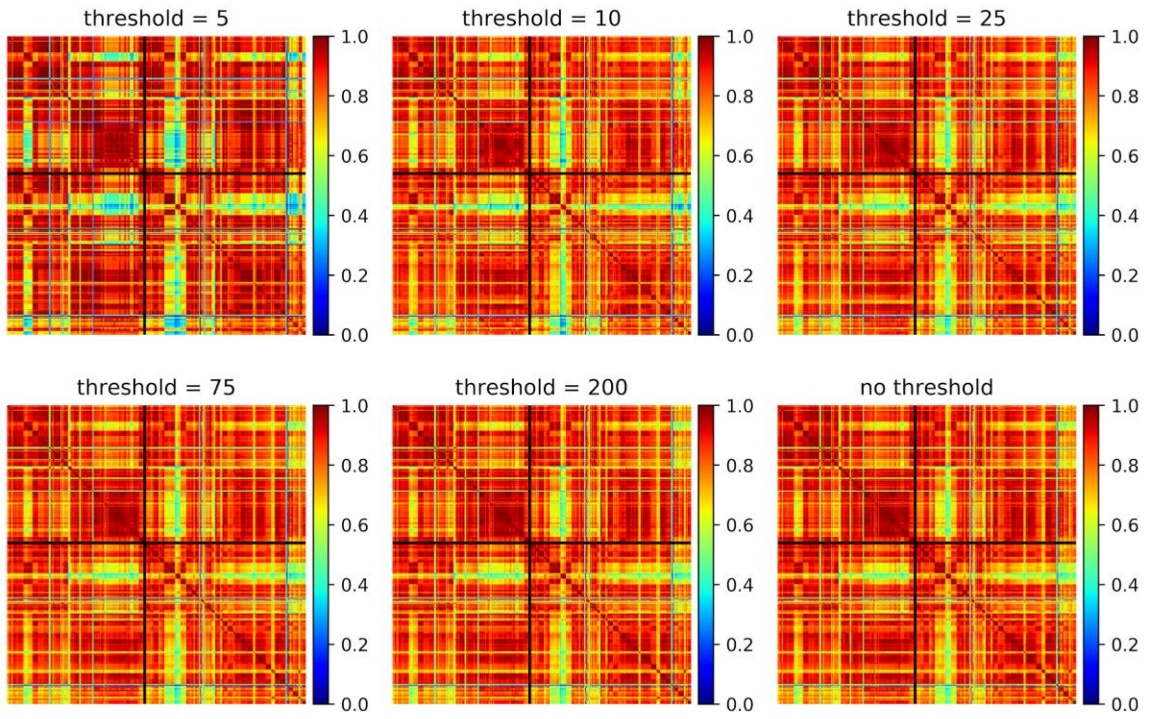

Figure S3. SSM for spectra sorted by diagnoses (and by measurement time inside each diagnosis) with different filtering thresholds (the amount of the most intensive peaks in each spectrum). Spectra measured under following conditions: positive mode, low-resolution, 100-2000 m/z. Black lines separate the astrocytoma and glioblastoma samples (top left - astrocytoma sector, bottom right - glioblastoma sector).

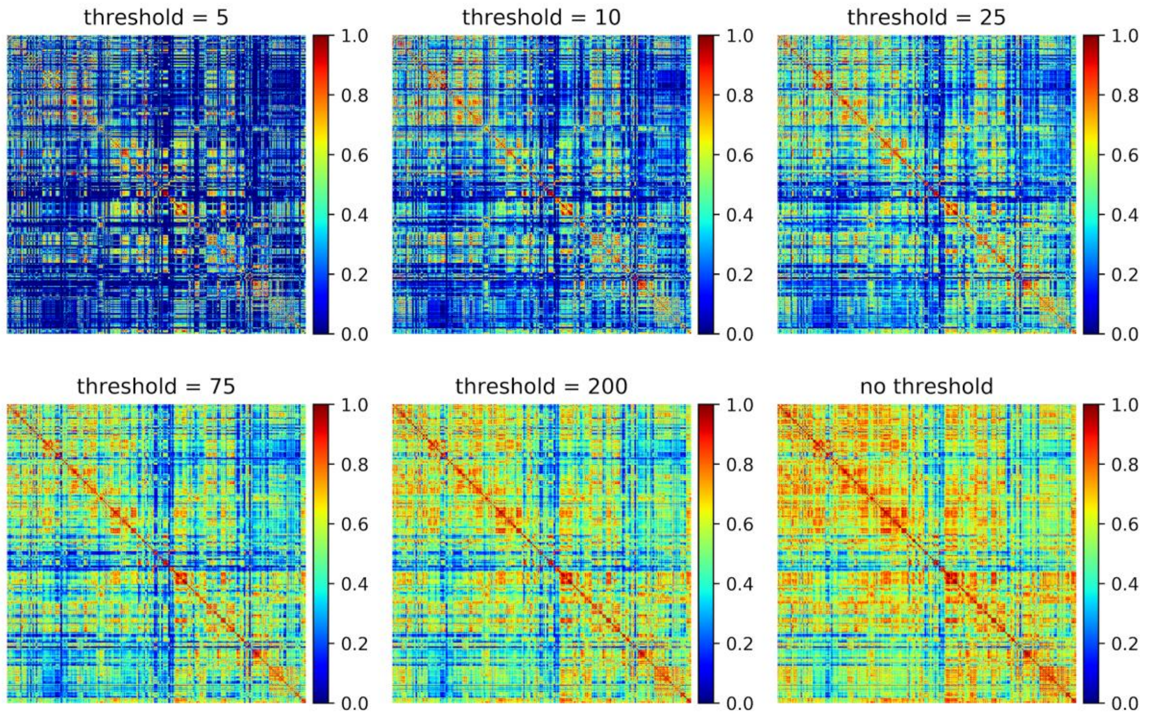

Figure S4. SSM for spectra sorted by measurement time with different filtering thresholds - the amount of the most intensive peaks in each spectrum. Spectra measured under following conditions: negative mode, low-resolution, 100-2000 m/z.

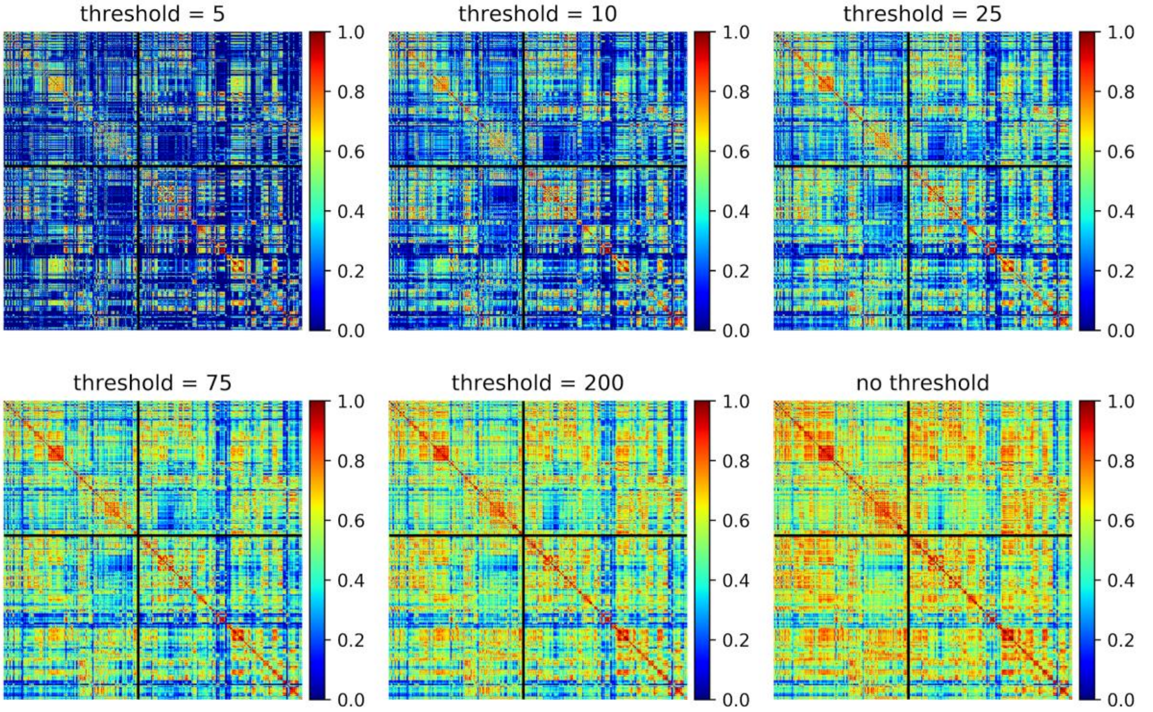

Figure S5. SSM for spectra sorted by diagnoses (and by measurement time inside each diagnosis) with different filtering thresholds (the amount of the most intensive peaks in each spectrum). Spectra measured under following conditions: negative mode, low-resolution, 100-2000 m/z. Black lines separate the astrocytoma and glioblastoma samples (top left - astrocytoma sector, bottom right - glioblastoma sector).

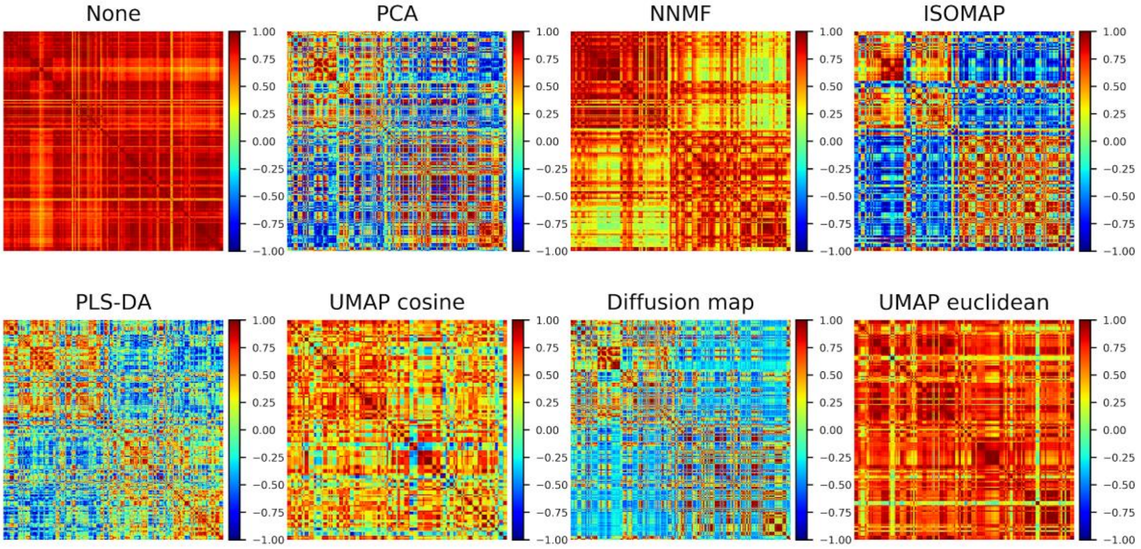

Figure S6. SSM for raw spectra and after dimensionality reduction with 5 left components. Sorted by measurement time. Spectra measured under following conditions: positive mode, low-resolution, 100-2000 m/z.

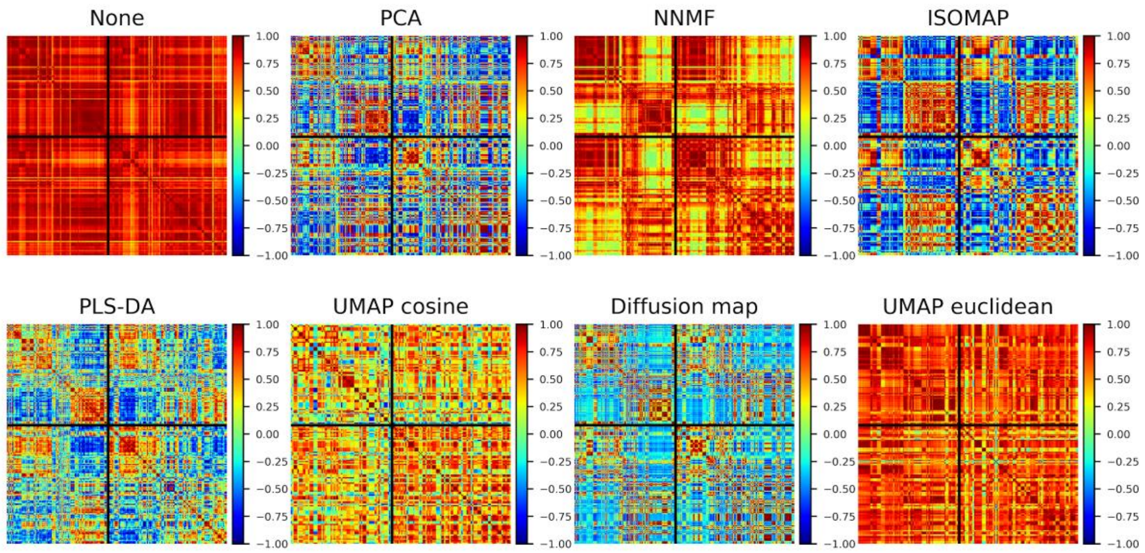

Figure S7. SSM for raw spectra and after dimensionality reduction with 5 left components. Sorted by diagnoses (and by measurement time inside each diagnosis). Spectra measured under following conditions: positive mode, low-resolution, 100-2000 m/z. Black lines separate the astrocytoma and glioblastoma samples (top left - astrocytoma sector, bottom right - glioblastoma sector).

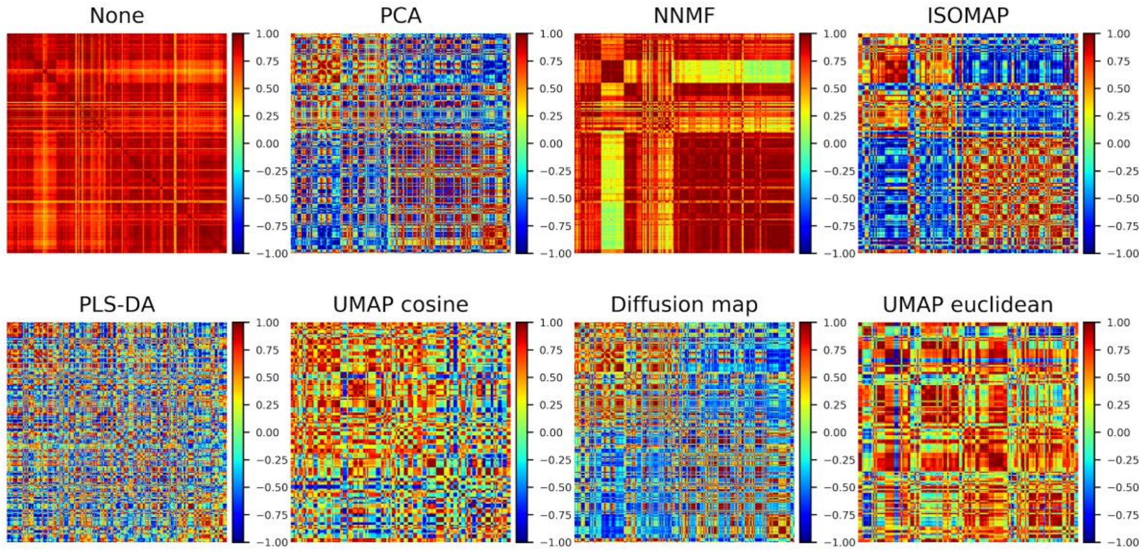

Figure S8. SSM for raw spectra and after dimensionality reduction with 3 left components. Sorted by measurement time. Spectra measured under following conditions: positive mode, low-resolution, 100-2000 m/z.

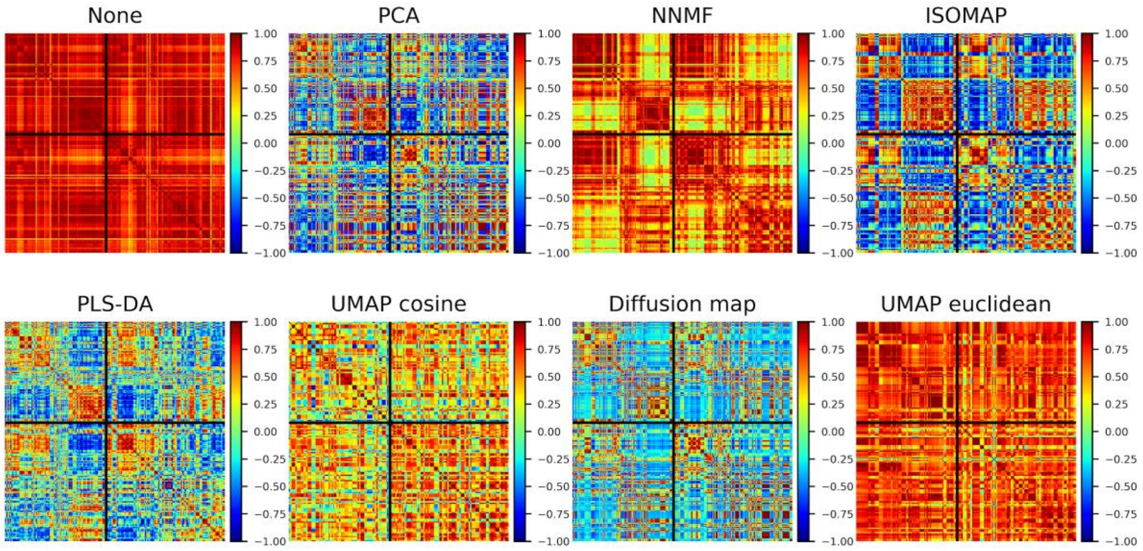

Figure S9. SSM for raw spectra and after dimensionality reduction with 3 left components. Sorted by diagnoses (and by measurement time inside each diagnosis). Spectra measured under following conditions: positive mode, low-resolution, 100-2000 m/z. Black lines separate the astrocytoma and glioblastoma samples (top left - astrocytoma sector, bottom right - glioblastoma sector).

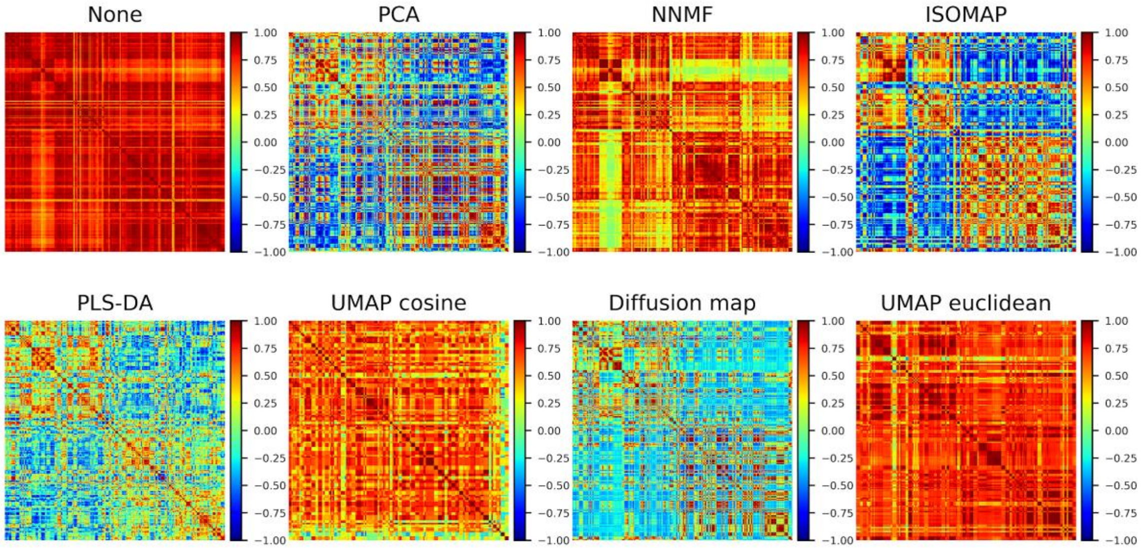

Figure S10. SSM for raw spectra and after dimensionality reduction with 7 left components. Sorted by measurement time. Spectra measured under following conditions: positive mode, low-resolution, 100-2000 m/z.

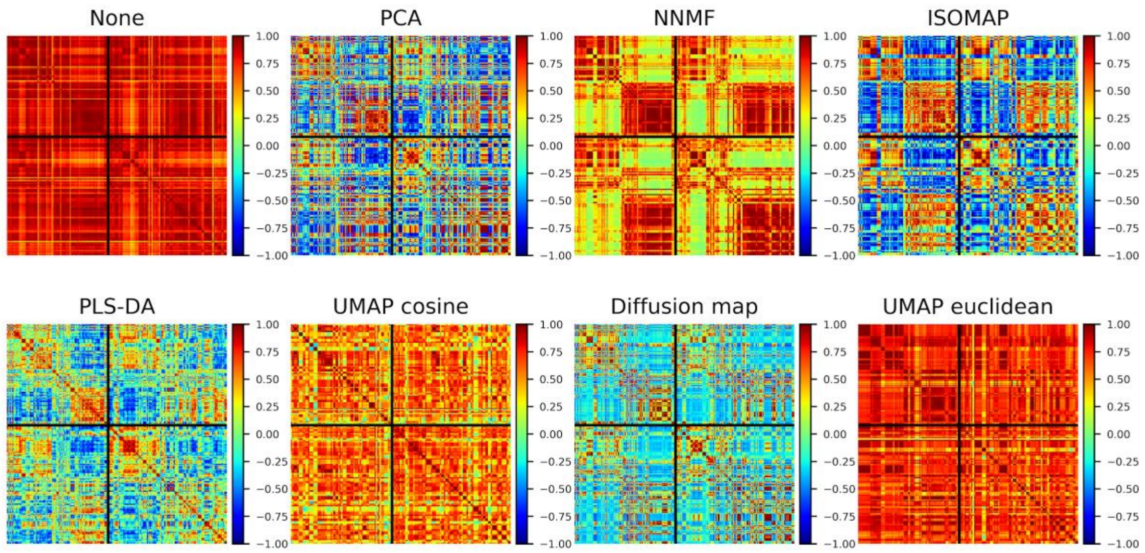

Figure S11. SSM for raw spectra and after dimensionality reduction with 7 left components. Sorted by diagnoses (and by measurement time inside each diagnosis). Spectra measured under following conditions: positive mode, low-resolution, 100-2000 m/z. Black lines separate the astrocytoma and glioblastoma samples (top left - astrocytoma sector, bottom right - glioblastoma sector).

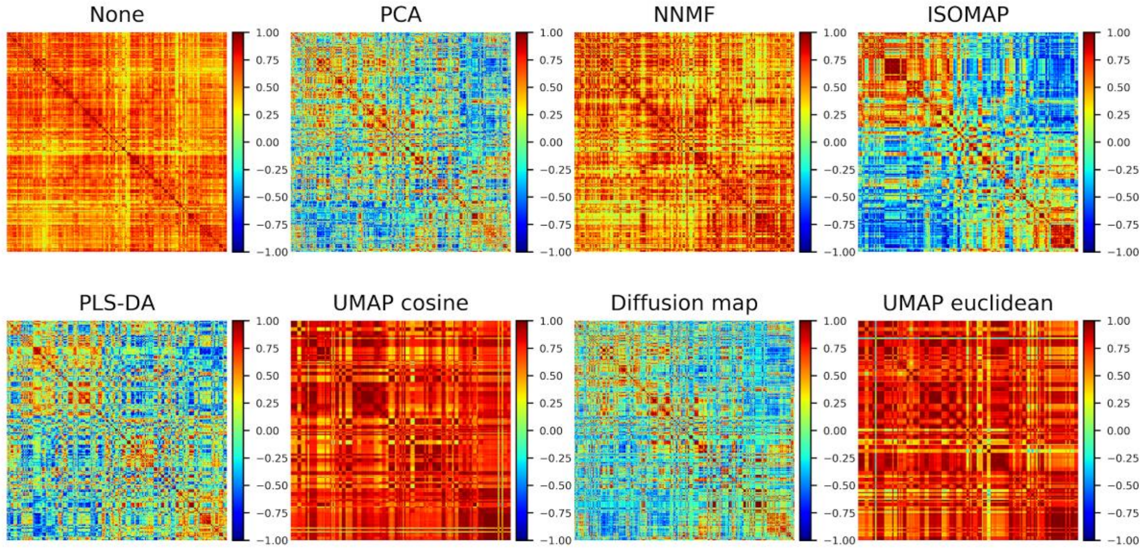

Figure S12. SSM for raw spectra and after dimensionality reduction with 5 left components. Sorted by diagnoses (and by measurement time inside each diagnosis). Spectra measured under following conditions: negative mode, low-resolution, 100-2000 m/z. Black lines separate the astrocytoma and glioblastoma samples (top left - astrocytoma sector, bottom right - glioblastoma sector).

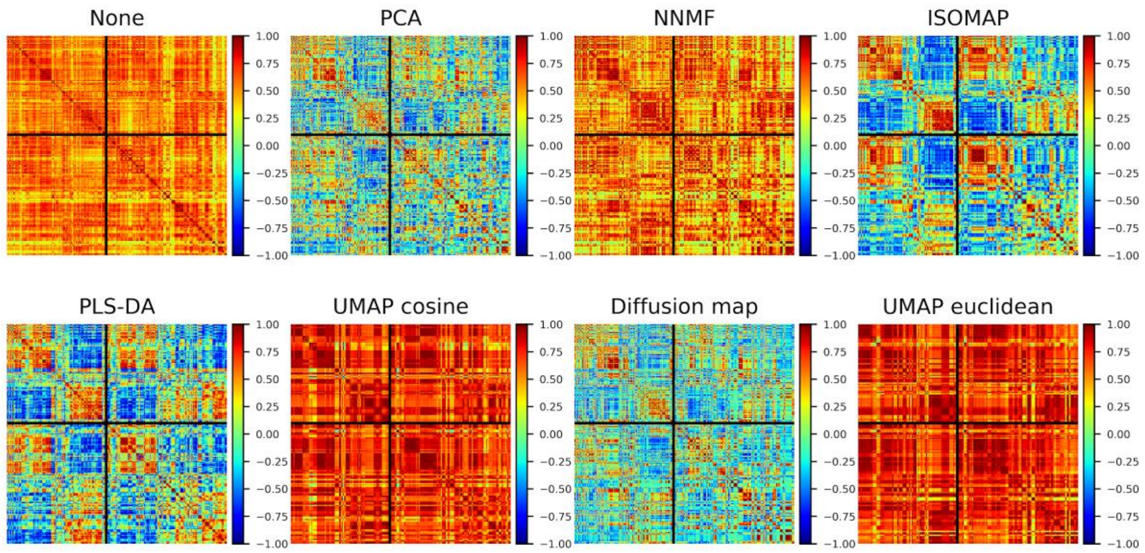

Figure S13. SSM for raw spectra and after dimensionality reduction with 5 left components. Sorted by diagnoses (and by measurement time inside each diagnosis). Spectra measured under following conditions: negative mode, low-resolution, 100-2000 m/z. Black lines separate the astrocytoma and glioblastoma samples (top left - astrocytoma sector, bottom right - glioblastoma sector).

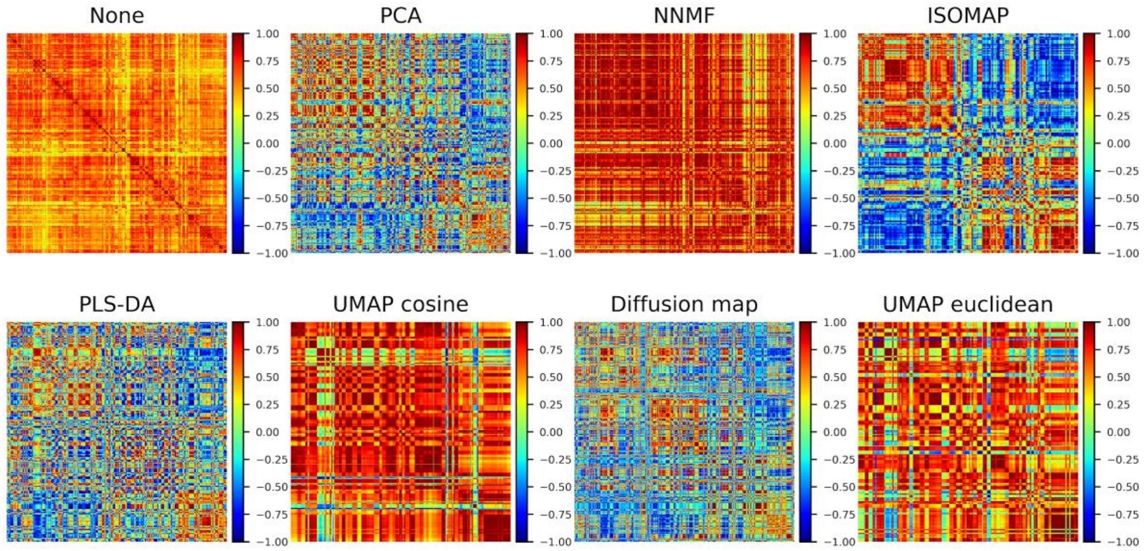

Figure S14. SSM for raw spectra and after dimensionality reduction with 3 left components. Sorted by diagnoses (and by measurement time inside each diagnosis). Spectra measured under following conditions: negative mode, low-resolution, 100-2000 m/z. Black lines separate the astrocytoma and glioblastoma samples (top left - astrocytoma sector, bottom right - glioblastoma sector).

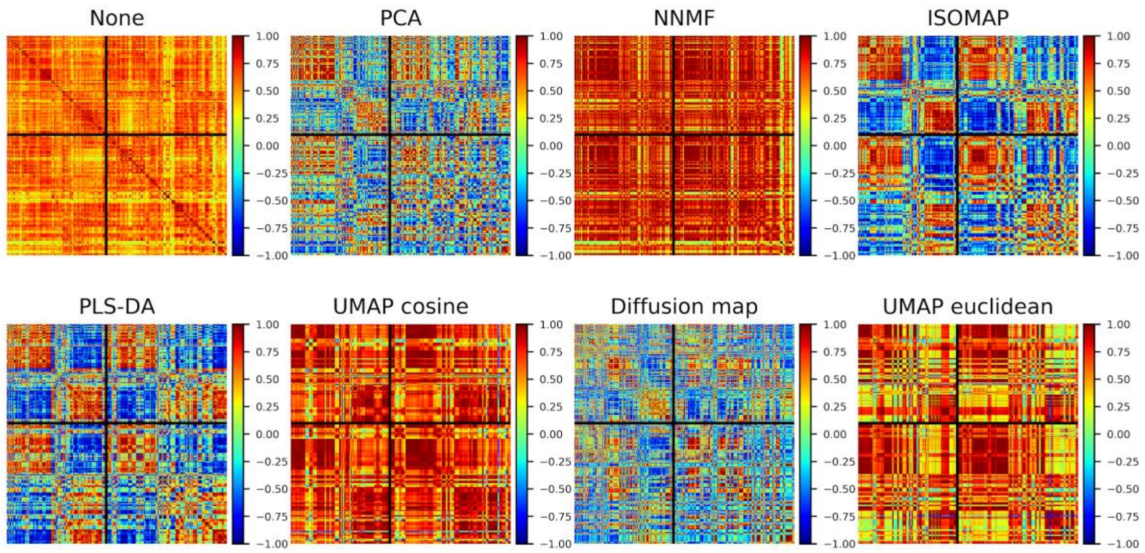

Figure S15. SSM for raw spectra and after dimensionality reduction with 3 left components. Sorted by diagnoses (and by measurement time inside each diagnosis). Spectra measured under following conditions: negative mode, low-resolution, 100-2000 m/z. Black lines separate the astrocytoma and glioblastoma samples (top left - astrocytoma sector, bottom right - glioblastoma sector).

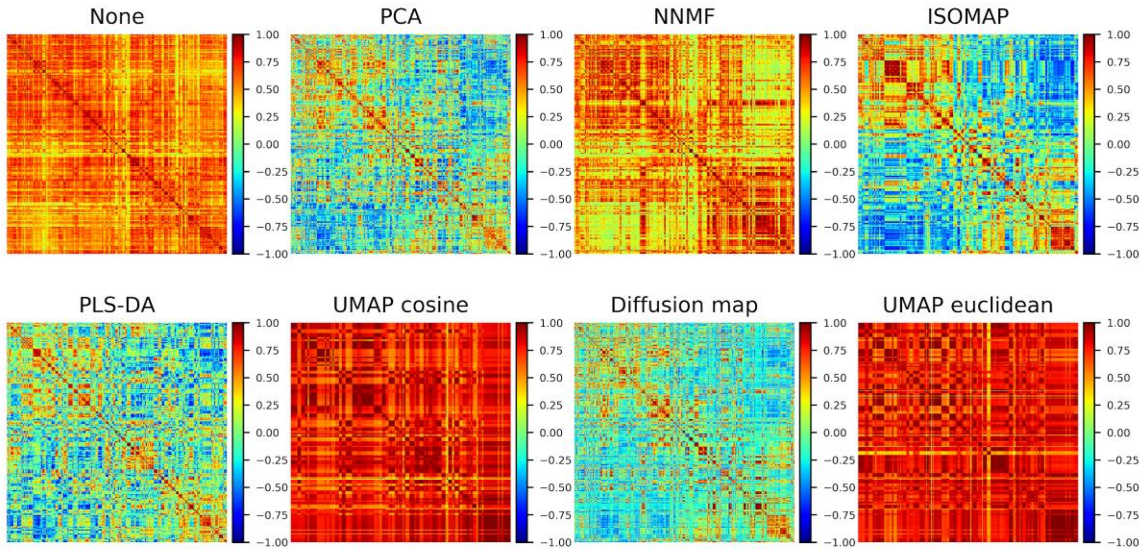

Figure S16. SSM for raw spectra and after dimensionality reduction with 7 left components. Sorted by diagnoses (and by measurement time inside each diagnosis). Spectra measured under following conditions: negative mode, low-resolution, 100-2000 m/z. Black lines separate the astrocytoma and glioblastoma samples (top left - astrocytoma sector, bottom right - glioblastoma sector).

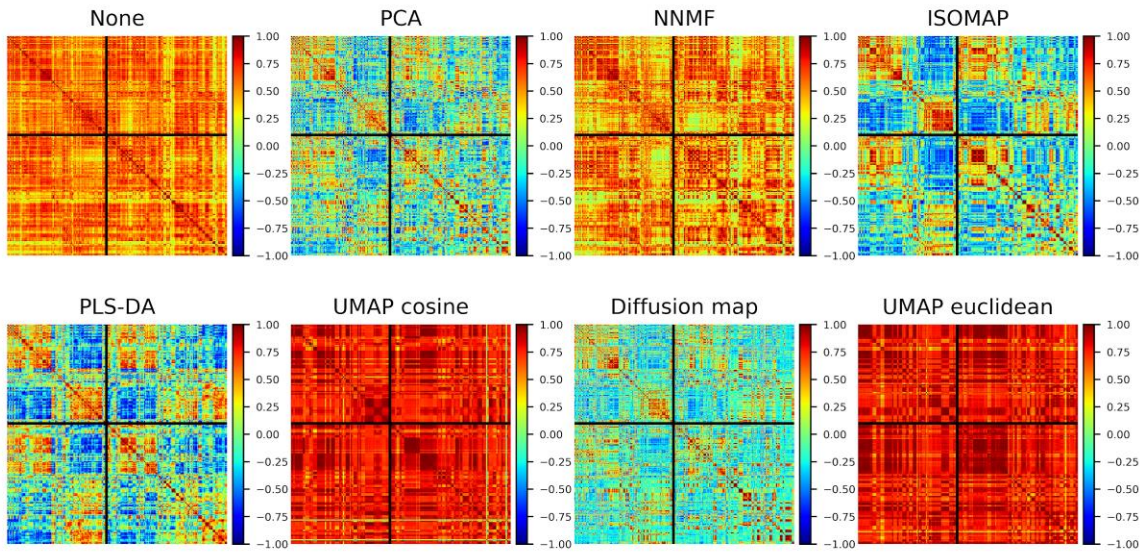

Figure S17. SSM for raw spectra and after dimensionality reduction with 7 left components. Sorted by diagnoses (and by measurement time inside each diagnosis). Spectra measured under following conditions: negative mode, low-resolution, 100-2000 m/z. Black lines separate the astrocytoma and glioblastoma samples (top left - astrocytoma sector, bottom right - glioblastoma sector).

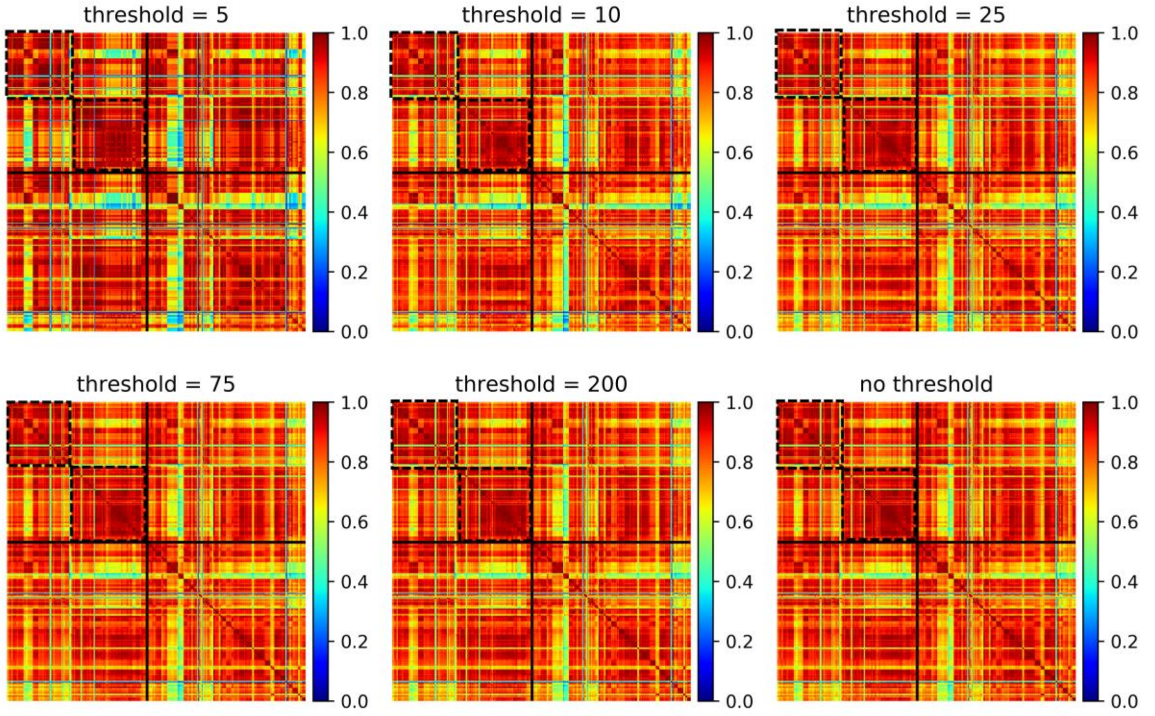

Figure S18. SSM for spectra sorted by diagnoses (and by measurement time inside each diagnosis) with different filtering thresholds (the amount of the most intensive peaks in each spectrum). Spectra measured under following conditions: positive mode, low-resolution, 100-2000 m/z. Black lines separate the astrocytoma and glioblastoma samples (top left - astrocytoma sector, bottom right - glioblastoma sector). Dashed squares point visually separated structures in astrocytoma data.

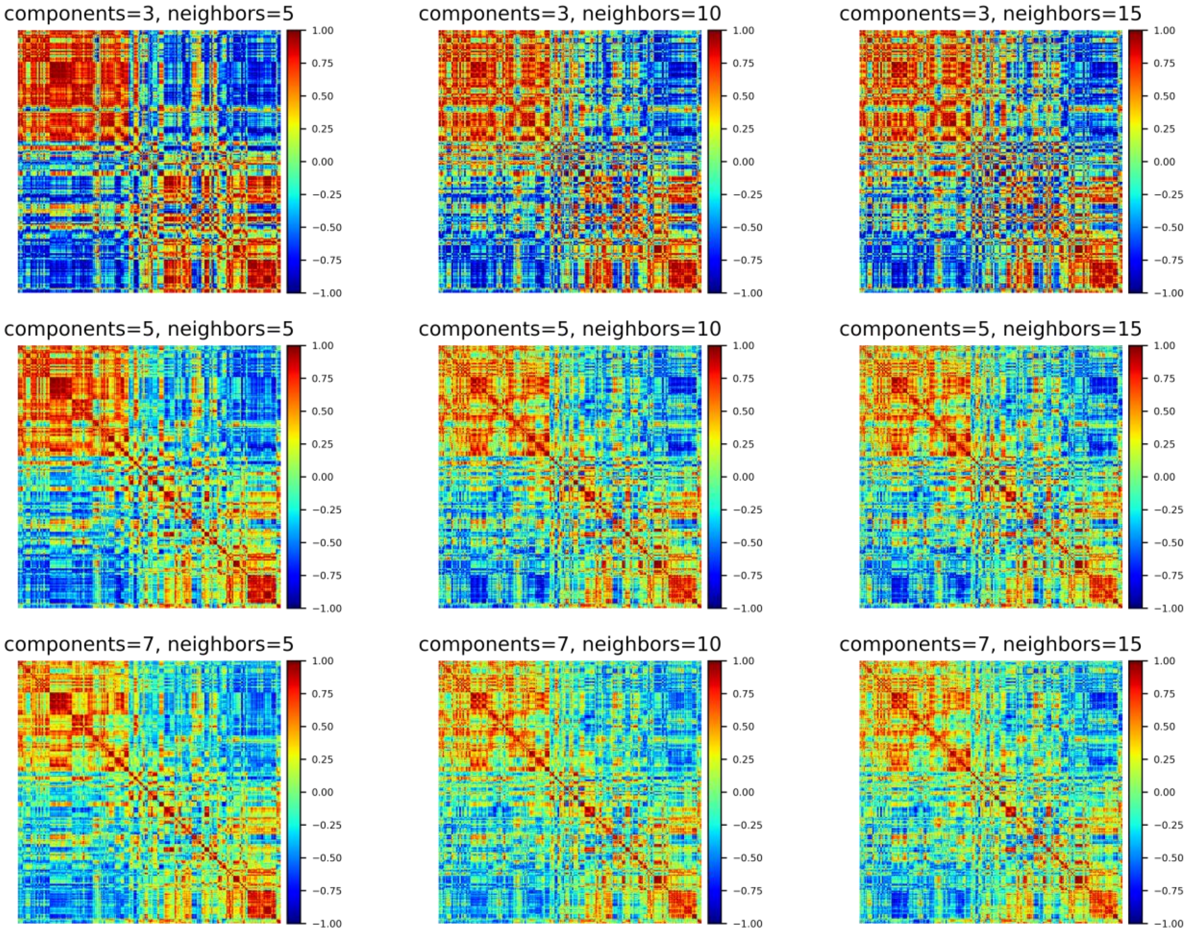

Figure S19. SSM for spectra sorted by measurement time compressed with Isomap. Different numbers of components and nearest neighbors are presented. Spectra measured under following conditions: negative mode, low-resolution, 100-2000 m/z.

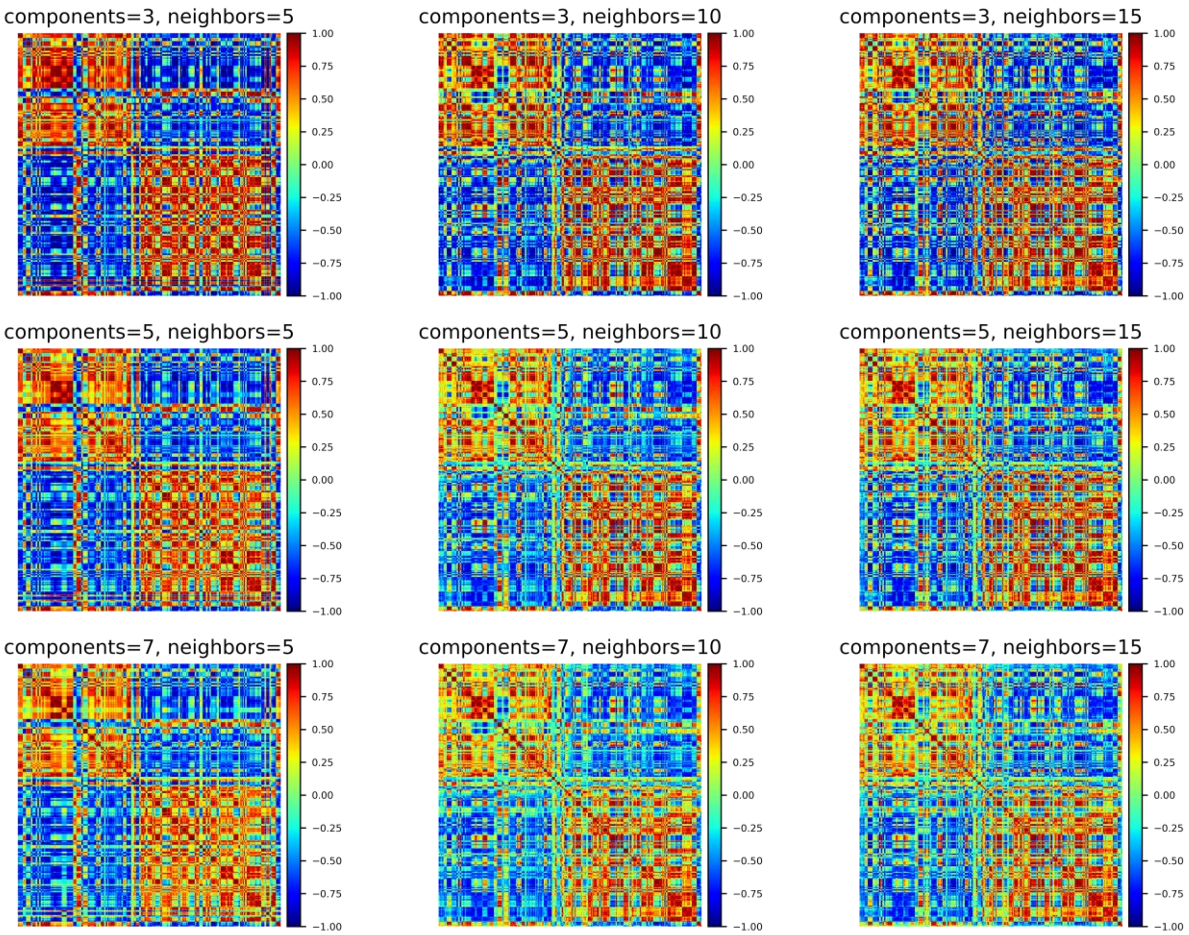

Figure S20. SSM for spectra sorted by measurement time compressed with Isomap. Different numbers of components and nearest neighbors are presented. Spectra measured under following conditions: positive mode, low-resolution, 100-2000 m/z.

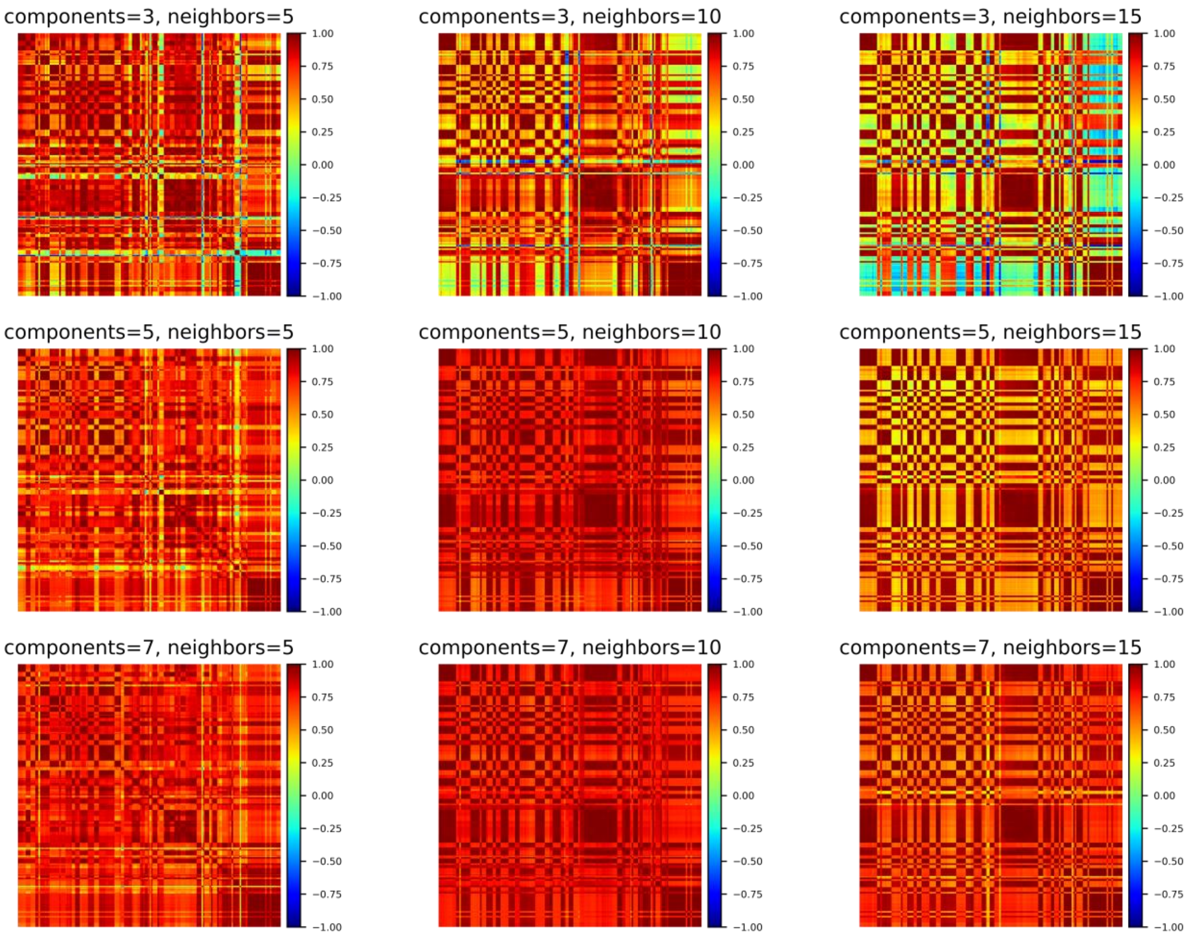

Figure S21. SSM for spectra sorted by measurement time compressed with UMAP. Different numbers of components and nearest neighbors are presented. Spectra measured under following conditions: negative mode, low-resolution, 100-2000 m/z.

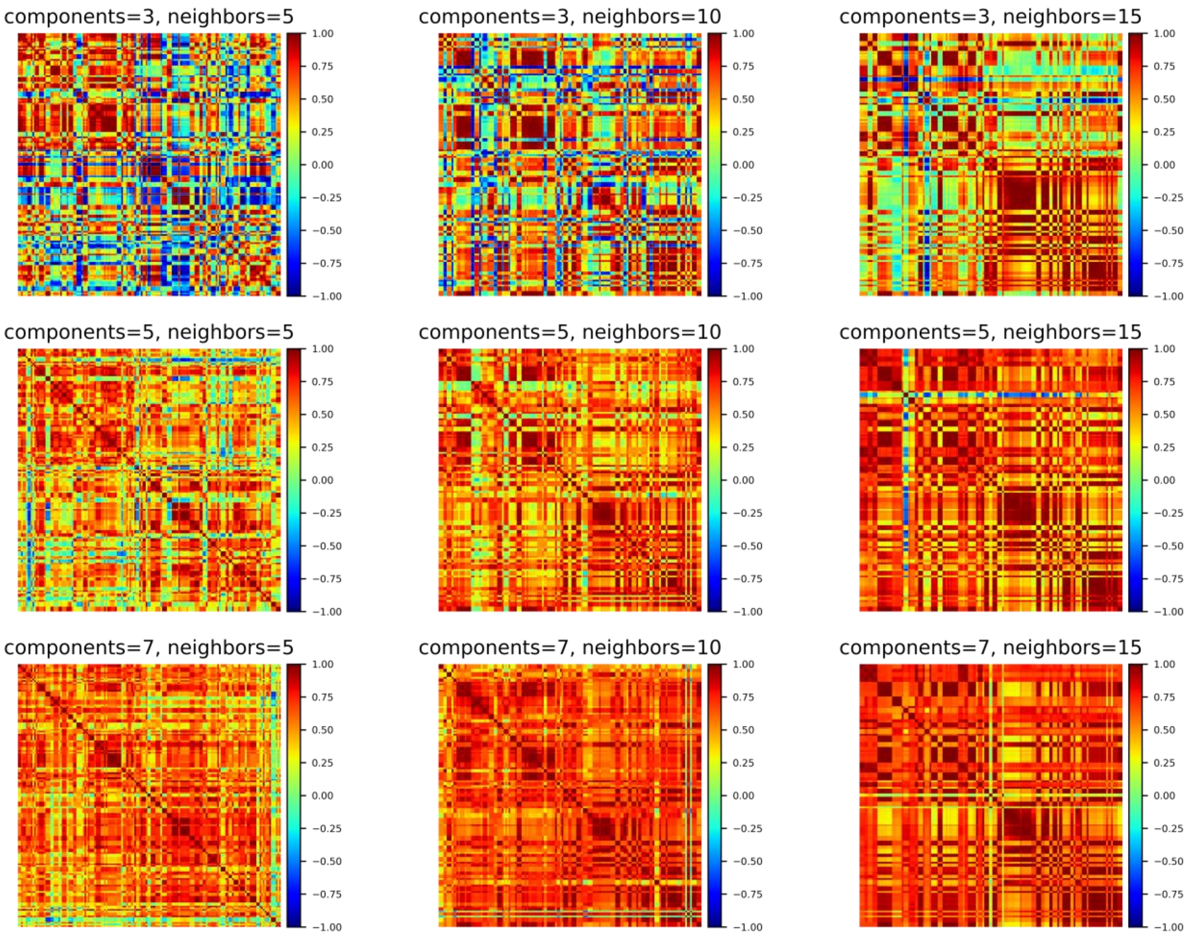

Figure S22. SSM for spectra sorted by measurement time compressed with UMAP. Different numbers of components and nearest neighbors are presented. Spectra measured under following conditions: positive mode, low-resolution, 100-2000 m/z.
